# Resources, support and integration as influencing factors in the implementation of blended therapy in psychiatric inpatient care

**DOI:** 10.1192/j.eurpsy.2025.1431

**Published:** 2025-08-26

**Authors:** W. Kawohl, N. Gupta

**Affiliations:** 1 Clienia Schlössli AG, Oetwil am See, Zurich; 2 Psychiatry, Psychotherapy and Psychosomatics, University of Zurich, Zurich, Switzerland; 3 UNIC Medical School, University of Nicosia, Nicosia, Cyprus; 4 Clienia Schlössli AG, Oetwil am See, Switzerland

## Abstract

**Introduction:**

Blended therapy (BT) is defined as the use of digital tools combined with traditional face-to-face therapy. Despite growing evidence concerning BT in outpatient settings, there is not much data on BT in inpatient care. To our knowledge, this is one of the first studies to investigate the implementation of BT in the routine care of psychiatric inpatients.

**Objectives:**

Our objective was to identify factors potential barriers and facilitating factors influencing the implementation of BT in psychiatric inpatient settings.

**Methods:**

We used a retrospective, exploratory qualitative research design and developed a semi-structured interview to collect our data. This enabled us to gain insights into the experiences of healthcare staff and patients. Participants were asked to reflect on the following themes we had pre-defined: resources, support, integration and sequential BT.

**Results:**

Therapists cited work overload with inadequate time adjustments and lack of ongoing training to sustain the use of BT in the long term as common barriers. A key finding of this study from the therapists’ perspective was also the importance of a clearly defined concept and setting for successful implementation. Patients valued the availability of satisfactory instructions for use, integration by therapists and a flexible setting. There were mixed opinions on sequential BT, with therapists mentioning in particular a lack of resources for patient care after discharge.

**Conclusions:**

In summary, the results show that therapists reported more barriers in contrast to patients’ perceptions. This highlights that while understanding the patient’s perspective is important, healthcare professionals play the central role in implementing and maintaining BT.

**Disclosure of Interest:**

None Declared

